# ﻿Comparative chromosome studies in species of subtribe Orchidinae (Orchidaceae)

**DOI:** 10.3897/compcytogen.v15.i4.75990

**Published:** 2021-12-17

**Authors:** Alessio Turco, Antonella Albano, Pietro Medagli, Robert P. Wagensommer, Saverio D’Emerico

**Affiliations:** 1 Dept. of Biological and Environmental Sciences and Technologies, University of Salento, Strada Provinciale Lecce-Monteroni, 73100 Lecce, Italy University of Salento Lecce Italy; 2 Dept. of Biology, Via E. Orabona 4, “Aldo Moro” University of Bari, 70125 Bari, Italy University of Bari “Aldo Moro Bari Italy; 3 “Aldo Moro” University of Bari, 70125 Bari, Italy University of Bari "Aldo Moro" Bari Italy

**Keywords:** *Anacamptis*, C-banding, Cytotaxonomy, FISH, *Himantoglossum*, Karyotype, *Ophrys*, *Serapias*

## Abstract

In our study, FISH mapping using 18S-5.8S-25S rDNA and 5S rDNA sequences was performed for the first time on *Ophrystenthredinifera* Willdenow, 1805, *Serapiasvomeracea* (Burman f., 1770) Briquet, 1910 and *Himantoglossumhircinum* (Linnaeus, 1753) Sprengel, 1826. A detailed study was also performed on *O.tenthredinifera* using Giemsa-staining, silver-staining, CMA fluorescence banding and fluorescence *in situ* hybridisation (FISH) with rDNA probes. We analysed two subspecies, i.e. O.tenthrediniferasubsp.neglecta (Parlatore, 1860) E.G. Camus, 1908 and O.tenthrediniferasubsp.grandiflora (Tenore, 1819) Kreutz, 2004 by the traditional Feulgen method and constructed the karyotype. The cytotaxonomic implications for both taxa are also discussed. In *Himantoglossumhircinum*, FISH and silver staining highlighted differences in the number of two rDNA families (35S and 5S) with respect to *Barliarobertiana* (Loiseleur-Deslongchamps, 1807) Greuter, 1967. In addition, fluorescence *in situ* hybridisation was also applied to diploid (2*n* = 2*x* = 36) and triploid (2*n* = 3*x* = 54) *Anacamptismorio* (Linnaeus, 1753) R.M. Bateman, Pridgeon et M.W. Chase, 1997. As far as we are aware, this is the first case of autotriploidy observed in *A.morio*.

## ﻿Introduction

Over the years, various karyological aspects including ploidy level, total length of the chromosome set, symmetry indices and amount of nuclear DNA (Siljak-Yakovlev and Peruzzi 2012) have proved to be useful tools for studying cytotaxonomy and for understanding chromosome evolution (Stace 2000; Guerra 2012; Ilnicki 2014; Sharma and Mukai 2015), as have other studies of structural variation looking at chromosomal features (e.g. secondary constrictions, AgNors and rDNA sites). In this context, these studies help to elucidate phylogenetic relationships between taxa. For example, analysis of differences in karyotype asymmetry have been shown to be good indicators of chromosomal diversification in terms of size and morphology within a group (Levitsky 1931; Stebbins 1971; Peruzzi and Eroğlu 2013; Astuti et al. 2017).

These cytogenetic studies have also played an important role in describing the main features in the systematics and phylogeny of orchids, based on both chromosomal analysis by traditional techniques (D’Emerico 2001 and references therein) and analyses of the structural variation of chromosomes (e.g. Giemsa C-banding, FISH, etc.) (Schwarzacher and Schweizer 1982; D’Emerico et al. 1999; D’Emerico et al. 2001; Moscone et al. 2007; Lan and Albert 2011).

The subtribe *Orchidinae* Vermeulen, 1977 comprises about 35 genera of mostly terrestrial orchids (Pridgeon et al. 2001), 15 of which occur in Italy (GIROS 2016; Bartolucci et al. 2018; Martellos et al. 2020). In this context, *Ophrys* Linnaeus, 1753 is probably the richest in species, many of which are endemic to restricted areas and are more or less threatened with extinction (Wagensommer et al. 2020; Turco et al. 2021). From a cytogenetic point of view, the chromosome numbers of at least 23 of these genera have been investigated by various researchers (D’Emerico 2001 and references therein). On the other hand, detailed investigations of the morphology of *Orchidinae* chromosomes have only been performed on the genera *Anacamptis* Richard, 1817, *Chamorchis* Richard, 1817, *Dactylorhiza* Necker ex Nevski, 1935, *Himantoglossum* Sprengel, 1826, *Neotinea* Reichenbach f., 1852, *Ophrys* Linnaeus, 1753, *Orchis* Linnaeus, 1753, *Platanthera* Richard, 1817, and *Serapias* Linnaeus, 1753 (D’Emerico 2005). Comparison of plant karyotypes using conventional cytological techniques can contribute to taxonomy and can provide insight into genome organisation and evolution in various genera (Turco et al. 2018).

The first systematic study of the karyomorphology of *Orchidinae* in Europe was undertaken by Bianco et al. (1988, 1989), who described the karyotypes of six different *Ophrys* species. In cytogenetic studies, in addition to the study of morphological chromosome features by traditional techniques, banding techniques with Giemsa and fluorochromes have also been used. These techniques have revealed variations in heterochromatin content in the chromosomal complements of some groups of *Orchidinae* (D’Emerico et al. 1996, 2000; D’Emerico et al. 2002a, 2002b; Pellegrino et al. 2000; D’Emerico et al. 2005; Baumann et al. 2012; Turco et al. 2020).

Molecular cytogenetic techniques such as fluorescence *in situ* hybridisation (FISH), used to identify repetitive sequence families and their distribution in plant chromosomes, have proven to be powerful tools for characterising chromosomes and investigating taxonomic relationships in plant groups (Maluszynska and Heslop-Harrison 1993a, 1993b; Galasso et al. 1995, 1997; Zoldos et al. 1999). D’Emerico et al. (2001) reported the physical distribution of 18S-5.8S-25S and 5S rDNA sequences in the chromosomes of five *Orchidinae* taxa for the first time.

Seeking to increase our knowledge and acquire data on the karyology and systematics of *Orchidinae*, we used FISH and other techniques to study the karyotypes and heterochromatin distribution of *Ophrystenthredinifera* s.l. and three other *Orchidinae*, i.e. *Himantoglossumhircinum* (Linnaeus, 1753) Sprengel, 1826, *Serapiasvomeracea* (Burman f., 1770) Briquet, 1910 and *Anacamptismorio* (Linnaeus, 1753) R.M. Bateman, Pridgeon et M.W. Chase, 1997, specifically their distribution of 18S-5.8S-25S and 5S rDNA loci, in order to elucidate their importance in the plants’ systematics and evolution.

## ﻿Methods

The taxa examined and their collection sites are shown in Table 1. Mitotic chromosomes were observed in tissues of immature ovaries. A total of fifteen individuals of *Ophrystenthredinifera* Willdenow, 1805 and five *Himantoglossumhircinum* were first analysed by Feulgen and C-banding methods. For these two species, at least ten metaphases were examined and the karyotype was constructed from well-spread metaphase plates. Four well-spread metaphase plates were then examined with the FISH technique. In addition, four specimens of *Anacamptismorio* and four of *Serapiasvomeracea* were analysed by the FISH technique and five metaphase plates were selected.

**Table 1.** Specimens of taxa analysed in this study.

Taxon	Provenance	Collector
Ophrystenthrediniferasubsp.grandiflora	Sicily, Francavilla di Sicilia (Messina); Niscemi (Caltanissetta)	Bartolo et Pulvirenti
Ophrystenthrediniferasubsp.neglecta	Apulia, Cassano Murge (Bari); Torre Canne (Brindisi), Gargano promontory (Foggia)	D’Emerico et Medagli
*Himantoglossumhircinum*	Apulia, Cassano Murge (Bari)	D’Emerico et Medagli
*Serapiasvomeracea*	Apulia, Adelfia (Bari)	D’Emerico
*Anacamptismorio*	Apulia, Cassano Murge (Bari)	D’Emerico

Immature ovary tissues were pre-treated with 0.3% colchicine at room temperature for 2 h. For Feulgen staining they were fixed for 5 min in 5:1:1:1 (v/v) absolute ethanol, chloroform, glacial acetic acid and formalin. Hydrolysis was performed at 20 °C in 5.5 N HCl for 20 min (Battaglia 1957a, b). The material was then stained in freshly prepared Feulgen stain. For C-banding, immature ovaries were fixed in 3:1 (v/v) ethanol–glacial acetic acid and stored in the deep-freeze for up to several months. Subsequently, they were squashed in 45% acetic acid; coverslips were removed by the dry ice method and the preparations air-dried overnight. Slides were then immersed in 0.2 N HCl at 60 °C for 3 min, thoroughly rinsed in distilled water and then treated with 4% Ba(OH)2 at 20 °C for 4 min. After thorough rinsing they were incubated in 2× SSC at 60 °C for 1 h. The stain used was 3–4% Giemsa (BDH) at pH 7.

For DAPI (4–6-diamidino-2-phenylindole) staining, ovaries were treated as for C-banding and stained using a buffered DAPI solution (0.6 mg/mL) for 5 min. after which they were rinsed and mounted in 1:1 (v/v) buffer and glycerol. For chromomycin A3 (CMA) staining, slides were stained with 0.5 mg/mL CMA for 1 h and mounted in 1:1 (v/v) McIlvaine’s pH 7.0 buffer–glycerol. For identification of the nucleolus, AgNO3 precipitation was used (Lacadena and Cermeno 1985).

For *Ophrystenthredinifera* Willd., 1805, chromosome measurements were performed using the freeware MicroMeasure 3.3 (http://www.colostate.edu/Depts/Biology/MicroMeasure). Chromosome pairs were identified and arranged on the basis of length. The nomenclature used for describing karyotype composition followed Levan et al. (1964). Karyotype morphometric characters were evaluated by calculating the haploid complement, while the karyotype asymmetry indices M_CA_ (Mean Centromeric Asymmetry) and CV_CL_ (Coefficient of Variation of Chromosome Length) were used for the evaluation of karyotype asymmetry. Moreover, CV_CI_ (Coefficient of Variation of the Centromeric Index) was used to evaluate heterogeneity in the position of the centromeres (Paszko 2006, Zuo and Yuan 2011, Peruzzi and Eroğlu 2013).

For fluorescence *in situ* hybridisation, the ribosomal sequences 18S-5.8S-25S (pTa71 - red signals) and 5S (pTa794 - green signals) were used as probes. Clone pTa71 was labelled with rhodamine-4-dUTP by nick translation, while pTa794 was labelled with digoxigenin-11-dUTP using polymerase chain reaction. The former contains a 9kb *EcoBl* repeat unit of 18S-5.8S-25S rDNA and intergenic spacer regions, isolated from *Triticumaestivum* Linnaeus, 1753 (Gerlach and Bedbrook 1979), and the latter corresponds to a complete 410 bp 5S gene unit, containing the 5S gene and intergenic spacer regions, isolated from *Triticumaestivum* (Gerlach and Dyer 1980). Pre-treatment of slides and the FISH procedure followed the protocol in Heslop-Harrison et al. (1991). The chromosomes and DNA probes were denatured together at 70 °C for 5 min and hybridisation was performed at 37 °C overnight. After hybridisation, the coverslips were removed in 2× SSC at room temperature and then washed thoroughly for 10 min in 20% (v/v) formamide in 0.1× SSC at 42 °C to remove sequences with less than 85% homology; the slides were then incubated in immunoflorescent reagents.

For detection of the digoxigenin-labelled probe, the slides were equilibrated in 4× SSC/0.1% (v/ v) Tween 20 and blocked in 5% (w/v) bovine serum albumin in 4× SSC/0.1% (v/v) Tween 20 for 5 min. Slides were incubated with sheep anti-digoxigenin antibody conjugated with FITC in a moist chamber at 37 °C for 1 h. The slides were washed in 4× SSC/Tween 20 3× 5 min and subsequently counterstained with DAPI prior to observation. They were finally mounted in antifade solution AF1 (Citifluor) and examined with a Leitz epifluorescence microscope with single and triple band pass filters. The resulting images were processed with freeware image-editing software, applying the functions to the whole image.

## ﻿Results

**Figure 1.**
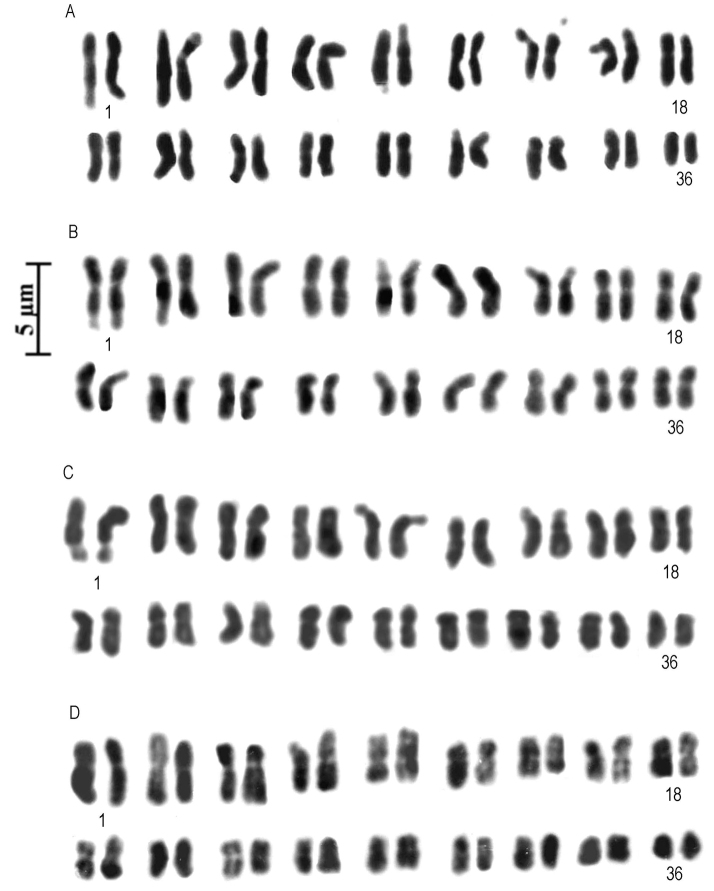
**A, B** Diploid karyotype of Ophrystenthrediniferasubspneglecta**C** diploid karyotype of *O.tenthredinifera* subsp. *grandiflora***D** diploid karyotype of *Himantoglossumhircinum*.

**Figure 2.**
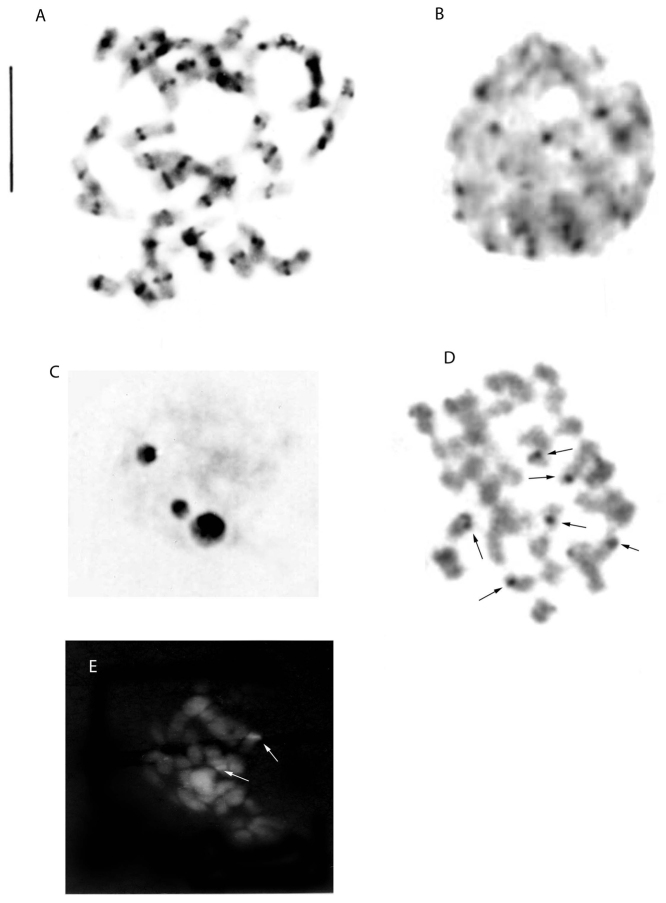
*Ophrystenthredinifera***A** Giemsa C-banding metaphase plate **B** Giemsa C-banding, interphase nucleus **C** silver staining, interphase nucleus **D** silver staining, mitotic metaphase **E** CMA staining, mitotic metaphase. Arrows indicate NOR sites. Scale bar: 5 µm.

**Figure 3.**
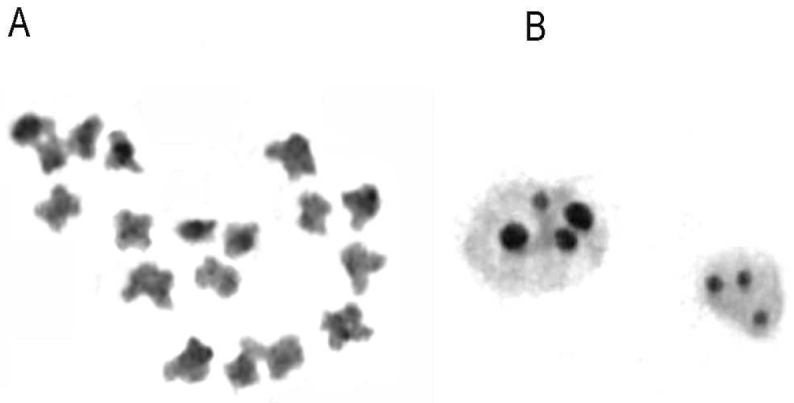
*Himantoglossumhircinum***A** Giemsa C-banding, haploid metaphase *n* = 18 **B** silver staining, interphase nucleus.

**Figure 4.**
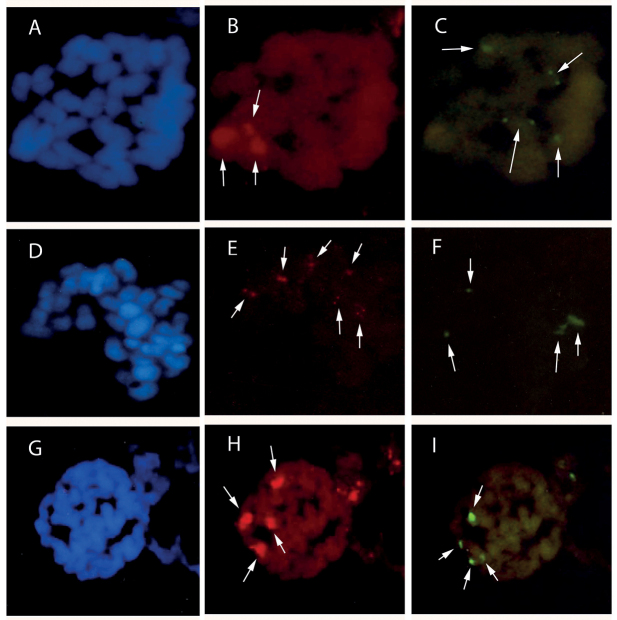
In situ hybridisation applied to chromosomes of orchid species. Blue DAPI staining shows chromosomal DNA (**A, D, G**, respectively *Ophrystenthredinifera*, *Serapiasvomeracea* and *Himantoglossumhircinum*); red and green signals show sites of hybridisation of 18S-25S rDNA and 5S rDNA respectively (**B, E, H, C, F, I**). Arrows indicate sites. *Ophrystenthredinifera* ( **B**) three 18S-25S rDNA sites and (**C**) four 5S rDNA sites. *Serapiasvomeracea* (**E**) six 18S-25S rDNA sites and (**F**) four 5S rDNA sites. *Himantoglossumhircinum* (**H**) four 18S-25S rDNA sites and (**I**) four 5S rDNA sites.

**Figure 5.**
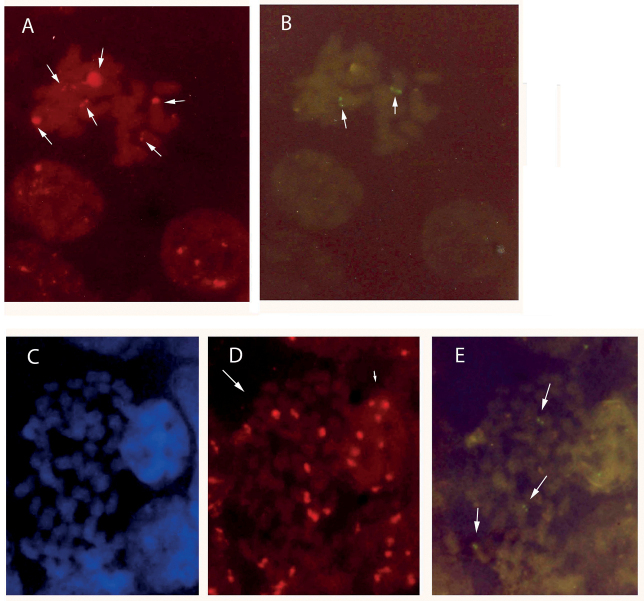
In situ hybridisation applied to chromosomes of *Anacamptismorio*. Blue DAPI staining shows chromosomal DNA ( **C**); red and green signals show sites of hybridisation of 18S-25S rDNA and 5S rDNA respectively (**A**, **D**, **B**, **E**). Arrows indicate sites. Mitotic metaphase 2*n* = 2*x* = 36 with (**A**) six 18S-25S rDNA sites and (**B**) two 5S rDNA sites. Mitotic metaphase 2*n* = 3*x* = 54 with (**D**) nine 18S-25S rDNA sites and (**E**) three 5S rDNA sites. Interestingly, a large number of 18S-5.8S-25S rDNA sites (9) were observed in interphase nuclei (small arrow).

The chromosomes of the studied species are shown in Figs 1–5. Unfortunately, in the analysed species it is rather difficult to obtain good metaphasic plates for FISH staining. Therefore, we considered only the visible signals with the pTa71 and pTa794 probes to document sites rich in GC and AT. The cytogenetic analysis showed the following characteristics.

Genus *Ophrys*: Mitotic metaphases in *Ophrystenthredinifera* showed the chromosome number 2*n* = 2*x* = 36. We analysed two subspecies of *O.tenthredinifera*, O.tenthrediniferasubsp.neglecta (Parlatore, 1860) E.G. Camus, 1908 and O.tenthrediniferasubsp.grandiflora (Tenore, 1819) Kreutz, 2004, with the traditional Feulgen method and composed the karyotype. The results are shown in Table 2. The karyotypes of both subspecies were similar in terms of both the satellite pairs and the asymmetry indices. It is possible to observe four pairs of chromosomes, each with a satellite on the short arm (Figs 1A, B, C). The karyological formula shows 32 metacentrics plus 4 submetacentrics in both subspecies. The karyotype is the most symmetrical, having a low intrachromosomal asymmetry (M_CA_) index (12.44–13.29) and a low interchromosomal asymmetry (CV_CL_) index (16.56–16.83).

**Table 2.** Taxon, chromosome number, formula and morphometric parameters (mean ± SE) in *Ophrystenthredinifera*. MCA (Mean Centromeric Asymmetry), CVCL (Coefficient of Variation of Chromosome Length), CVCI (Coefficient of Variation of Centromeric Index). Chromosome abbreviations: m, metacentric; sm, submetacentric.

Taxon	Number of individuals	Chromosome number (2n)	Formula	M_CA_	CV_CL_	CV_CI_
Ophrystenthrediniferasubsp.grandiflora	5	36	32m + 4sm	12.44 ± 2.59	16.83 ± 0.84	8.43 ± 1.41
O.tenthrediniferasubsp.neglecta	10	36	32m + 4sm	13.29 ± 0.11	16.56 ± 0.88	10.80 ± 0.79

In *O.tenthredinifera*, C-banding showed the presence of centromeric heterochromatin, with a pair of chromosomes with a telomeric band. A large number of chromocentres were observed in interphase nuclei (Fig. 2A, B). The nucleolus organiser regions (NORs) revealed by Ag-NOR staining were located in the telomeric region of the third chromosome pair (Fig. 2D), evidence that the six rDNA sites were active. However, in interphase nuclei it was possible to count up to three nucleoli (Fig. 2C). Moreover, in *O.tenthredinifera*, CMA staining revealed a positive signal on the NOR-bearing pair only (Fig. 2E). FISH analyses with the pTa71 (18S-5.8S-25S) probe showed three signals (Fig. 4B), as revealed by the Ag-NOR staining in interphase nuclei (Fig. 2C). In addition, this species showed two pairs of 5S rDNA sites (Fig. 4C).

Genus *Serapias*: Mitotic metaphases in *Serapiasvomeracea* had 2*n* = 2*x* = 36 chromosomes. *In situ* hybridisation shows that there are three pairs of 18S-5.8S-25S rDNA sites (Fig. 4E). The 5S rDNA sequence was present on two pairs of chromosomes (Fig. 4F).

Genus *Himantoglossum*: All specimens of *Himantoglossumhircinum* had 2*n* = 2*x* = 36 chromosomes. The *H.hircinum* karyotype consists of 28m + 8sm. In *H.hircinum* a secondary constriction was seen in the short arm of pairs 4, 5 and 7 (Fig. 1D). C-banding revealed that some chromosomes in *H.hircinum* possess small amounts of centromeric and telomeric constitutive heterochromatin (Fig. 3A). In interphase nuclei it was possible to count up to four nucleoli (Fig. 3B). Moreover, FISH revealed the location of four 18S-5.8S-25S rDNA sites (Fig. 4H) and four 5S rDNA sites (Fig. 4I).

Genus *Anacamptis*: In diploid *Anacamptismorio* (2*n* = 2*x* = 36), silver nitrate staining in interphase nuclei counted up to four nucleoli. In this study, *in situ* hybridisation revealed the location of six 18S-5.8S-25S rDNA sites (Fig. 5A) and two 5S rDNA sites (Fig. 5B). Fluorescence *in situ* hybridisation in triploids (2*n* = 3*x* = 54) revealed nine 18S-5.8S-25S rDNA sites (Fig. 5D) and three 5S rDNA sites (5E).

## ﻿Discussion

This paper reports the physical locations of rDNA loci on the somatic chromosomes of *Ophrystenthredinifera*, *Serapiasvomeracea* and *Himantoglossumhircinum* for the first time. Our analyses showed 18S-5.8S-25S rDNA sites and 5S rDNA sites in triploid specimens of *Anacamptismorio*.

The chromosome numbers, karyotype asymmetry and heterochromatin content of spontaneous populations of *Ophrystenthredinifera* were determined. Mitotic metaphase plates showed 2*n* = 2*x* = 36 chromosomes in all studied populations of *O.tenthredinifera*, which confirms the karyological stability of this taxon throughout its area of distribution (Scrugli 1977; Bianco et al. 1991; Bernardos et al. 2003; D’Emerico et al. 2005). Bernardos et al. (2003) reported 2*n* = 38 + 4B and 2*n* = 38 chromosomes for *O.tenthredinifera* in Iberia and North Africa respectively, while 2*n* = 3*x* = 54 was reported by Bianco et al. (1991) in only one case. The first chromosome pair clearly shows a secondary constriction on the long arm, as observed in other works (D’Emerico et al. 2005; Deniz et al. 2017).

Regarding the infraspecific taxonomy of *Ophrystenthredinifera*, this study analysed two subspecies, namely O.tenthrediniferasubsp.neglecta, endemic to Sardinia and peninsular Italy from Tuscany to Calabria, and O.tenthrediniferasubsp.grandiflora, endemic to Sicily and southern Calabria (GIROS 2016). The present study showed few intraspecific karyotype variations between populations of O.tenthrediniferasubsp.neglecta and O.tenthrediniferasubsp.grandiflora. These data do not therefore support a separation of these two taxa, as suggested in the World Checklist of selected Plant Families (WCSP), http://wcsp.science.kew.org/home.do, by Hennecke and Galano (2020).

Silver nitrate staining in interphase nuclei showed three nucleoli, although some meristematic cells had one large nucleolus. Moscone et al. (1995) suggest that the maximum number of nucleoli per nucleus generally coincides with the maximum number of NORs detected with silver nitrate. However, the number may be lower, due to frequent nucleoli fusions.

The 18S-5.8S-25S rRNA genes are normally located on the nucleolus organizing secondary constriction and adjacent heterochromatin, of which the nucleolar organiser region (NOR) is constituted. Whereas 5S rDNA sites are exclusively detected by FISH, they do not form chromosome constrictions in metaphase chromosomes (Fuchs et al. 1998). *In situ* hybridisation shows that there are two pairs of 5S rDNA sites in *Ophrystenthredinifera*. However, in *O.tenthredinifera*, the pTa794 signals were not intense.

The two subspecies O.tenthrediniferasubsp.neglecta and O.tenthrediniferasubsp.grandiflora may be affected by the epigenetic effects of heterochromatic sequences present on chromosomes. Indeed, Paun et al. (2010) analysed three sibling allotetraploid orchid species differing radically in terms of their geographical and ecological contexts, and showed that ecological divergence in *Dactylorhiza* species is mostly due to epigenetic factors regulating gene expression in response to environmental stimuli. Unfortunately, in the genus *Ophrys*, as far as we know, no study of this kind has been conducted.

Previous cytological studies in *Serapiasvomeracea* have shown 2*n* = 2*x* = 36 chromosomes (Heusser 1938; Del Prete 1977; Mazzola et al. 1981; Bianco et al. 1987). This species shows a moderately asymmetrical karyotype consisting of mainly submetacentric chromosomes (D’Emerico et al. 1992). Giemsa C-banding analysis showed conspicuous bands in centromeric positions on many chromosomes, together with euchromatic telomeric regions (D’Emerico et al. 2000). In *Serapiasvomeracea* the 5S rDNA signals on one pair were much more intense than those on the other pair. The presence of a major site of 5S rDNA gene clusters could be regarded as further evidence of recent chromosomal restructuring (Abbo et al. 1994) of this species, reinforcing previous reports (D’Emerico et al. 2001 and references therein).

*Serapias* comprises about 25 species (Delforge 2016), and cytological studies have shown that most of them have 2*n* = 2*x* = 36 chromosomes (D’Emerico et al 2000; Bernardos et al. 2004; Bellusci and Aquaro 2008). Polyploidy has been observed in *S.lingua* Linnaeus, 1753 (Brullo et al. 2014), *S.olbia* Verguin, 1908, *S.gregaria* Godfrey, 1921 and *S.strictiflora* Welwitsch ex Veiga, 1887 (Bellusci and Aquaro 2008), all with 2*n* = 4*x* = 72 chromosomes. The karyotype of numerous species of the genus *Serapias* has been observed using the Giemsa technique, with interesting results. Indeed, C-banded somatic metaphase plates showed broad centromeric bands on almost all chromosomes where heterochromatin occupies most of the chromosome, leaving a euchromatic segment in a telomeric position (D’Emerico et al. 2000). The karyology of *Serapiaslingua* is interesting from the data obtained through conventional analyses alone, with numerous bivalents compared to the few tetravalents observed at metaphase I in EMC. Unfortunately, for this genus, the only data obtained with FISH in this study are reported for *S.vomeracea*.

*Himantoglossum* s.l. (including *Comperia* K. Koch, 1849 and *Barlia* Parlatore, 1860) is a group of species found in Portugal, Spain and across the Mediterranean region, including North Africa, the Aegean islands, Syria and Turkey, as well as the Crimea, the Caucasus and western and northern Iran (Wood 2001). The species *Himantoglossumhircinum* and *H.adriaticum* H. Baumann, 1978 have a chromosome number of 2*n* = 2*x* = 36. Ströhlein and Sundermann (1972) reported 2*n* = 2*x* = 30 in *Himantoglossumcomperianum* (Steven, 1829) P. Delforge, 1999, and Bernardos et al. (2006) reported 2*n* = 2*x* = 36 for *Himantoglossummetlesicsianum* (W.P. Teschner, 1982) P. Delforge, 1999. The chromosomal numbers of the other species of the genus such as *H.formosum* (Steven, 1813) K. Koch, 1849, *H.calcaratum* (Beck, 1887) Schlechter, 1927, *H.caprinum* Sprengel, 1826 and *H.montis-tauri* Kreutz et W. Lüders, 1997 (Bateman et al. 2017) are unknown. Cases of aneuploidy with 2*n* = 36+1B are known in both *H.hircinum* and *H.adriaticum* (Capineri and Rossi 1987; D’Emerico et al. 1993).

It is interesting to note that the World Checklist of selected Plant Families (WCSP) reports *Barliarobertiana* (Loiseleur-Deslongchamps, 1807) Greuter, 1967 as a synonym for *Himantoglossumrobertianum* (Loiseleur-Deslongchamps, 1807) P. Delforge, 1999. Furthermore, in the new classification based on morphological and molecular data (Sramkó et al. 2014), Bateman et al. (2017) place *Barliarobertiana* in the new subgenus Barlia (Parlatore, 1860) R.M. Bateman, Molnar et Sramkó, 2017 within the genus *Himantoglossum*.

Comparative investigations of *Himantoglossumhircinum* and *Barliarobertiana* show similar karyotype morphologies, with mainly metacentric chromosomes, low asymmetry and little constitutive heterochromatin. *H.hircinum* was found to have four nucleoli in interphase nuclei. Moreover, in situ hybridisation showed four 18S-5.8S-25S rDNA sites and four 5S rDNA sites. In contrast, double-target in situ hybridisation in *Barliarobertiana* revealed one pair of chromosomes carrying both the pTa794 and pTa71 signals on opposite arms (D’Emerico et al. 2001). In addition, interphase nuclei in *Barliarobertiana* had two nucleoli.

Giemsa C-banding and FISH yielded few data for *Himantoglossumhircinum* and *Barliarobertiana*, while for *H.adriaticum*, on which only the conventional Feulgen method was used, only the karyotype was established. As already mentioned, the asymmetry indices and karyological formulas of *Himantoglossumhircinum* and *Barliarobertiana* are so similar that it is hard to clearly distinguish between them. Furthermore, we did not obtain important data with Giemsa C-banding; the few discriminating data are visible only with silver staining and FISH. Therefore, in the future it will be useful to continue with the above analyses in order to obtain clarification regarding the phylogenetic relationships between *Barliarobertiana* and the other species of the genus *Himantoglossum*.

In *Anacamptismorio*, the chromosome number 2*n* = 2*x* = 36 is consistent with previous reports (D’Emerico et al. 1996 and references therein). The karyotype consists of 30m + 6sm. This species possesses the most symmetrical karyotype, comprising mainly metacentric chromosomes. Three satellited chromosomes were visible. In our study, neither the chromosomes nor the interphase nuclei of this species showed any differential reaction when stained with Giemsa or DAPI. Fluorescence *in situ* hybridisation mapping in diploid *Anacamptismorio* showed six 18S-5.8S-25S rDNA sites and two 5S rDNA sites. In contrast, in a previous paper, D’Emerico et al. (2001) reported four 18S-5.8S-25S rDNA sites and two 5S rDNA sites.

In this study we report analyses of a triploid individual of *Anacamptismorio* with chromosome number 2*n* = 3*x* = 54 for the first time. The same count has been reported in specimens of *A.coriophora* (Linnaeus, 1753) R.M. Bateman, Pridgeon et M.W. Chase, 1997, *A.laxiflora* (Lamarck, 1779) R.M. Bateman, Pridgeon et M.W. Chase, 1997 and *A.pyramidalis* (Linnaeus, 1753) Richard, 1817 (D’Emerico et al. 1992, 1993; Pegoraro et al. 2016, 2019; Doro 2020). Fluorescence *in situ* hybridisation mapping in this triploid showed nine 18S-5.8S-25S rDNA sites and three 5S rDNA sites.

## ﻿Conclusions

Fluorescence in situ hybridisation may authentically substantiate the genome structure and distribution of repetitive DNA families. In this context, our results provide new data on the cytogenetic differences between four genera within the Orchidinae and investigations of other species are expected to yield further insight. Moreover, these data constitute basic knowledge for facilitating the study of taxonomic relationships in other species of this subtribe. Some examples are given below.

In relation to the triploid individuals observed in the genus *Anacamptis* s.l., it is possible to add some interesting notes about *A.pyramidalis*, useful for other species where polyploid individuals have been observed. The species *A.pyramidalis* has 2*n* = 2*x* = 36, 54, 72 chromosomes (D’Emerico et al. 1992, 1993; Pegoraro et al. 2016, 2019). Recently the new species *A.berica* Doro, 2020 has been reported by Doro (2020), with 2*n* = 4x = 72 chromosomes. In both cases, polyploid species with 2*n* = 4*x* = 72 are referred to as autotetraploid, although Bianco et al. (1991) reported 36 bivalents with occasional quadrivalents at metaphase I in EMC. Giemsa C-banding has not yielded good results, but it would be interesting to analyse these species with other banding methods and FISH.

Also interesting from the karyological point of view are the *Neotinea* s.l. group, with 2*n* = 2*x* = 42 chromosomes, and the polyploid insular neoendemic *N.commutata* (Todaro, 1842) R.M. Bateman, 2003 with 2*n* = 4*x* = 84 (Mazzola et. al. 1981). An interesting result for *N.commutata* was reported by Pavarese et al (2013), who showed 42 bivalents at Metaphase I in EMC, hypothesising that the species arose from allopolyploidisation. In this case, no further data was obtained by the FISH method.

The *Orchis* s.s. group, with a chromosomal number of 2*n* = 2*x* = 42, is characterised by small chromosomes and a fairly complex structure. Polyploidy has been observed in *Orchiscanariensis* Lindley, 1835, *O.olbiensis* Reuter ex Grenier, 1860 and *O.patens* Desfontaines, 1799, with 2*n* = 4*x* = 84 chromosomes (Pellegrino et al. 2000; Bernardos et al. 2002; Bernardos et al. 2006). The *O.mascula* (Linnaeus, 1753) Linnaeus, 1755 complex, which includes the species *O.mascula*, *O.provincialis* Balbis ex Lamarck et Candolle, 1806, *O.pauciflora* Tenore, 1811 and *O.patens*, is still undergoing taxonomic evaluation. Cytogenetic analyses using differential banding methods based on Giemsa and fluorochromes such as DAPI and CMA3 have revealed a remarkable affinity between these species. In *O.mascula*, C-banded somatic metaphase chromosomes show distinctive heterochromatin distribution. In ten chromosome pairs of the complement, heterochromatin occupies most of the chromosomes, with euchromatin occupying only the telomeric region, while eleven pairs were euchromatic, characterised by the presence of thin centromeric bands. After staining with fluorochrome DAPI, the chromosomes of this species showed blocks of heterochromatin in telomeric and subtelomeric regions (D’Emerico et al. 2002a). The results reveal that the species of the *O.mascula* s.l. complex are cytogenetically different from those belonging to the remaining species of *Orchis* s.s. These differences open up interesting avenues of investigation regarding the involvement of heterochromatin in the evolutionary processes of these species. The presence of these chromosomal structures needs to be studied further, through these and other techniques.
